# Supplementary material for: Enhanced CAR-T activity against established tumors by polarizing human T cells to secrete interleukin-9
Source: Nat Commun. 2020 Nov 19;11:5902. doi: 10.1038/s41467-020-19672-2 (PMC7677397; doi:10.1038/s41467-020-19672-2)
Supplement: Supplementary file 1 — Supplementary Information [file 41467_2020_19672_MOESM1_ESM.pdf]

Supplementary Information

Enhanced CAR-T activity against established tumors by polarizing human T cells to secrete interleukin-9

Lintao Liu[1*], Enguang Bi[1*#], Xingzhe Ma[1*], Wei Xiong[1*], Jianfei Qian[1], Lingqun Ye[1], Pan Su[1], Qiang Wang[1], Liuling Xiao[1], Maojie Yang[1], Yong Lu[2], Qing Yi[1#]

Supplementary Figures 1-11

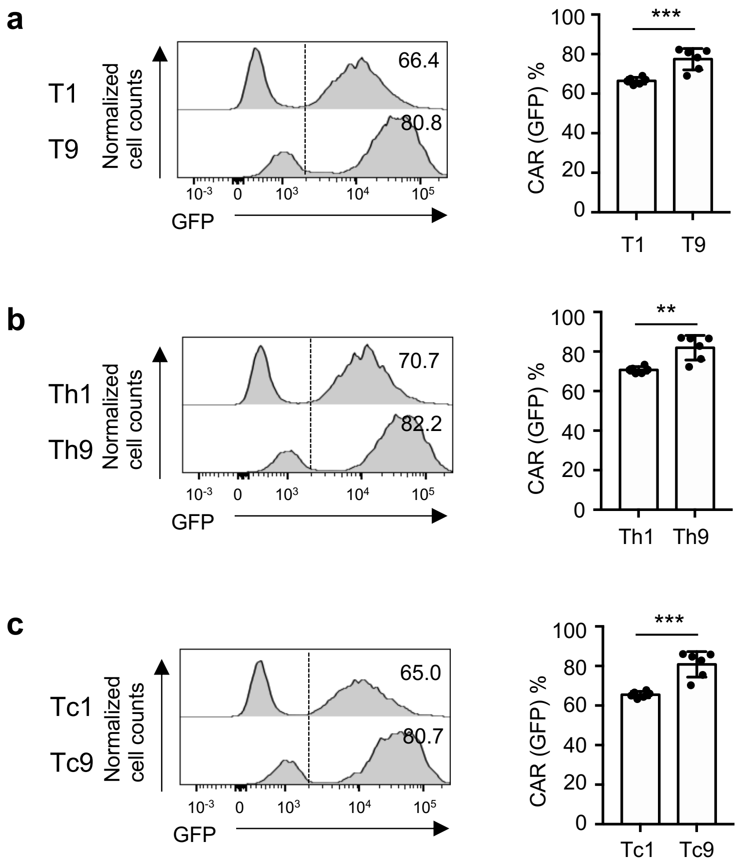

Supplementary Figure 1. CAR expression in CAR-T cells. CAR-T cells were generated and expanded for 16 days under Th1- or Th9-polarization condition. Percentage of GFP positive T cells was calculated in gated (**a**) $CD3^+$, (**b**) $CD3^+CD4^+$, or (**c**) $CD3^+CD8^+$ T cell populations. Data are presented as mean ± SD. n = 6 donors, *P < 0.05 and ***P < 0.001, two-sided Student's *t*-test. Source data are provided as a Source Data file.

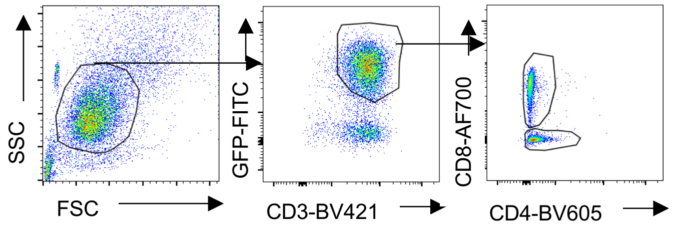

Supplementary Figure 2. Gating strategy for analyzing GFP$^+$CD3$^+$CD4$^+$ or GFP$^+$CD3$^+$CD8$^+$ CAR-T cells. This strategy was utilized to gate GFP$^+$CD3$^+$CD4$^+$ or GFP$^+$CD3$^+$CD8$^+$ CAR-T cells for analysis in this manuscript figures.

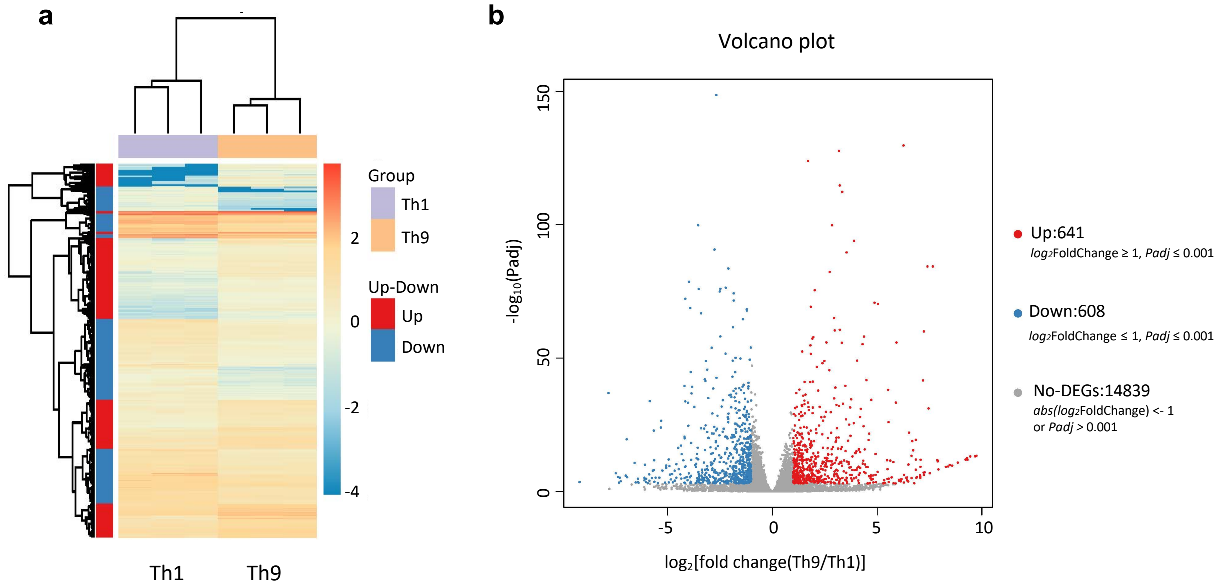

Supplementary Figure 3. Summary of DNBSeq analysis. **(a)** Global transcriptional profiles revealed by DNseq of purified CAR-T cells polarized and expanded for 16 days after lentivirus transduction. The heatmap shows the log2-fold change relative to the global average of the top upregulated and downregulated genes, with a cutoff of change in expression >2-fold and a p value < 0.05. (**b)** Volcano plot showing differentially expressed genes identified through RNA sequencing (Th1 CAR-T and Th9 CAR-T cells; n = 3 donors).

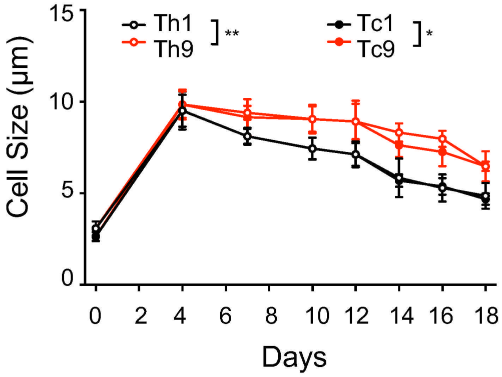

Supplementary Figure 4. Cells size of CAR-T cells. Size of CAR-T cells was measured by cell counters at indicated time points. T cell size during ex vivo expansion was monitored after anti-CD3/CD28 bead stimulation until day 18. Results are expressed as the mean T cell size (± SD). n = 4 donors, *P < 0.05 and **P < 0.01, two-way ANOVA. Source data are provided as a Source Data file.

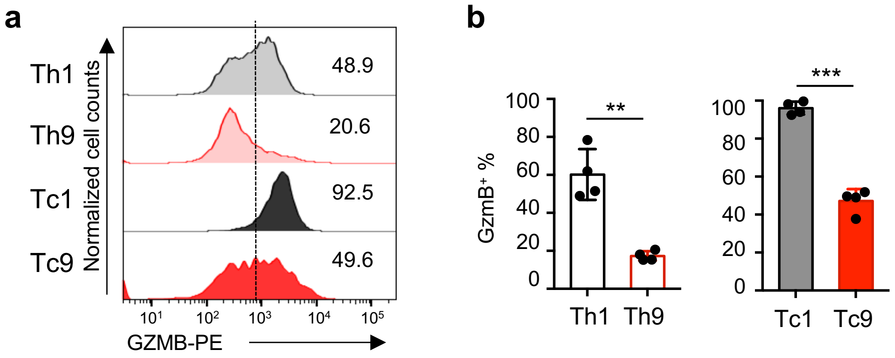

Supplementary Figure 5. GzmB expression in CAR-T cells. (**a**) Representative flow plots showing granzyme B expression in CAR-T cells on day 16 after in-vitro expansion. Cells were pre-gated for $GFP^+CD3^+CD8^+$ or $GFP^+CD3^+CD4^+$ T cells. (**b**) Sumarized results showing expression of granzyme B in CAR-T cells. Data are presented as mean ± SD (n = 4 donors, **P < 0.01 and ***P < 0.001, two-sided Student's *t*-test). Source data are provided as a Source Data file.

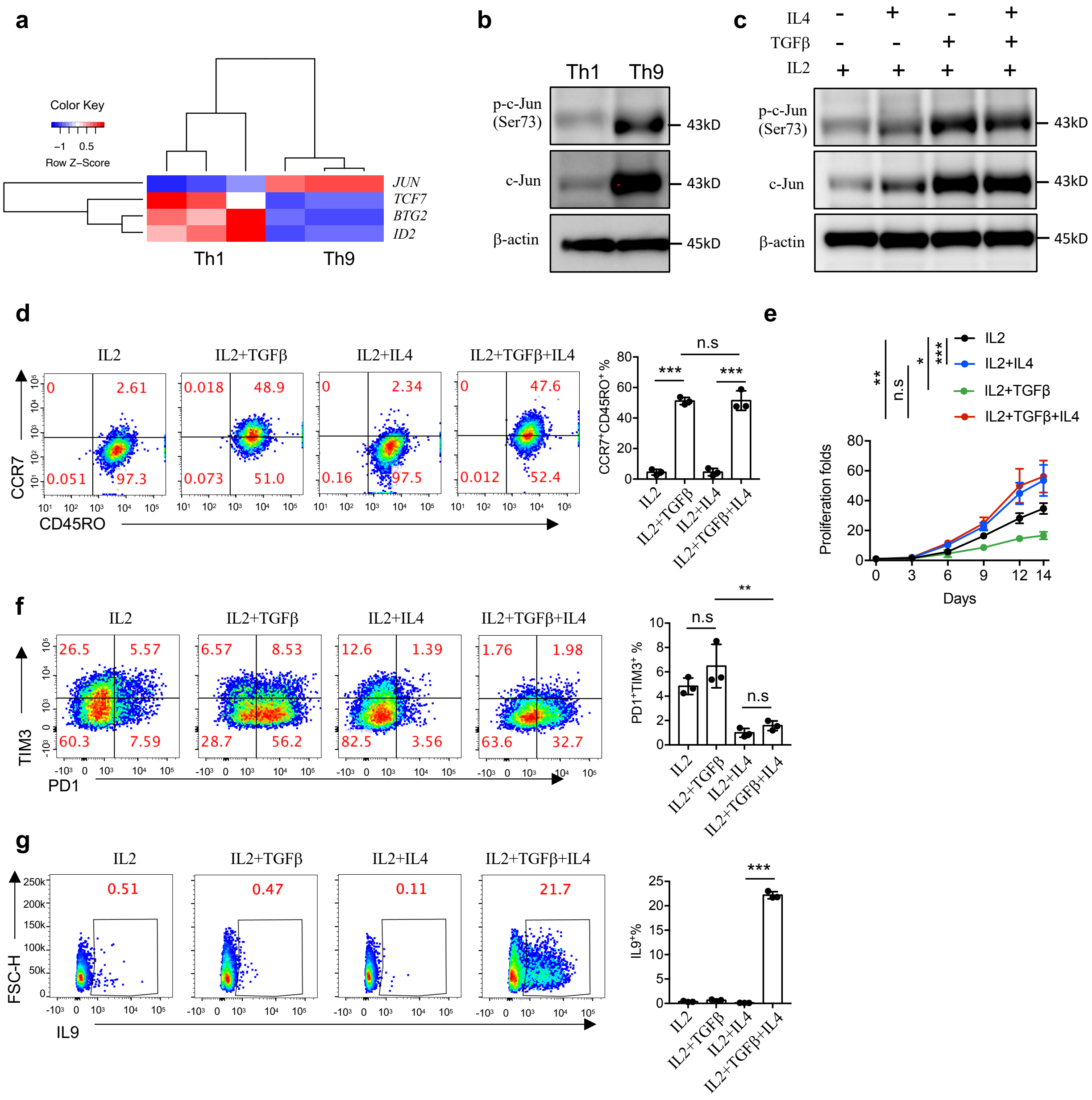

Supplementary Figure 6. IL4 and TGFβ act in synergy to dictate the phenotype of T9 CAR-T cells. (a) Heatmap illustrating the expression of genes of transcription factors in Th9 or Th1 CAR-T cells. (**b**) CAR-T cells were activated with anti-CD3/CD28 beads for 14 days, and the indicated proteins were analyzed by Western blot. CAR-T cells were activated with anti-CD3/CD28 beads for 14 days under different polarization conditions, and assayed for (**c**)

expression of the indicated proteins by Western blot, (**e**) proliferation by trypan blue exclusion (n = 4 donors, two-way ANOVA), (**d**) memory markers (CCR7 and CD45RO) (n = 3 donors), (**f**) exhaustion markers (n = 3 donors), and (**g**) IL9 expression by flow cytometry (n = 3 donors). Data shown in **c** are representative of three independent experiments. Data are presented as mean ± SD, n.s= no significance, *P < 0.05, **P < 0.01, and ***P < 0.001, two-sided Student's *t*-test. Source data are provided as a Source Data file.

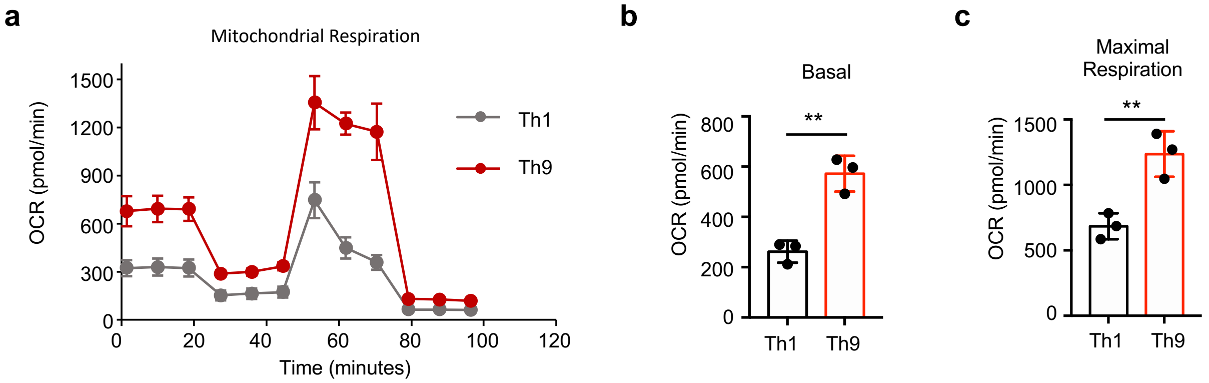

Supplementary Figure 7. **(a)** Oxygen consumption rates (OCRs), **(b)** basal OCR levels, and **(c)** maximum respiratory levels in human CD4 C AR-T cells 16 days after anti-CD3/CD28 beads stimulation. Experiments were performed with three biological replicates and data shown are representative of two independent experiments. Data are presented as mean ± SD. **P < 0.01, two-sided Student's *t*-test. Source data are provided as a Source Data file.

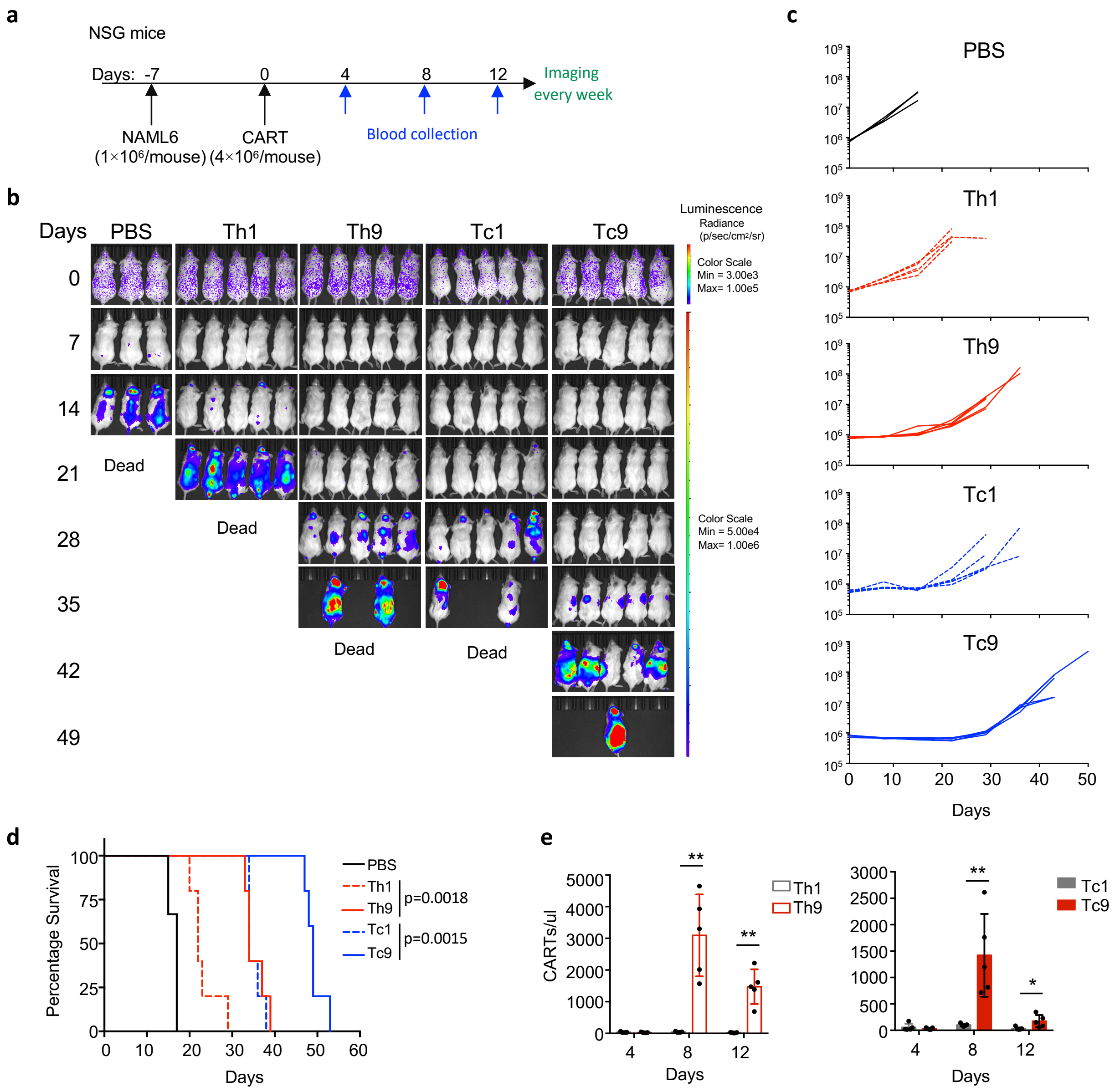

Supplementary Figure 8. Both CD4$^+$ Th9 and CD8$^+$ Tc9 CAR-T cells are able to exert strong antitumor activity against established tumor in vivo. (a) NSG mice were intravenously injected with 1 × 10^6 NALM6 cells. Seven days later, mice were randomly assigned to 5 groups and were infused intravenously with 4 × 10^6 CAR-T cells. **(b)** Tumor burden measured by bioluminescence at indicated days after CAR-T cell infusion. **(c)** Tumor burden (total flux) quantified by photons/s in mice treated with PBS or CAR-T cells at indicated

days after CAR-T cell infusion. **(d)** Kaplan-Meyer plot showing mouse survival. Exact P values from a log-rank test are shown for T1 versus T9 CAR-T cell-treated mice. **(e)** Total number of $CD3^+$ CAR-T cells in blood of treated mice at different days since CAR-T infusion. Summarized results (n = 5 mice) are shown. Data are presented as mean ± SD (n = 6 donors. **P < 0.01, *P < 0.05 and ***P < 0.001, two-sided Student's *t*-test). Source data are provided as a Source Data file.

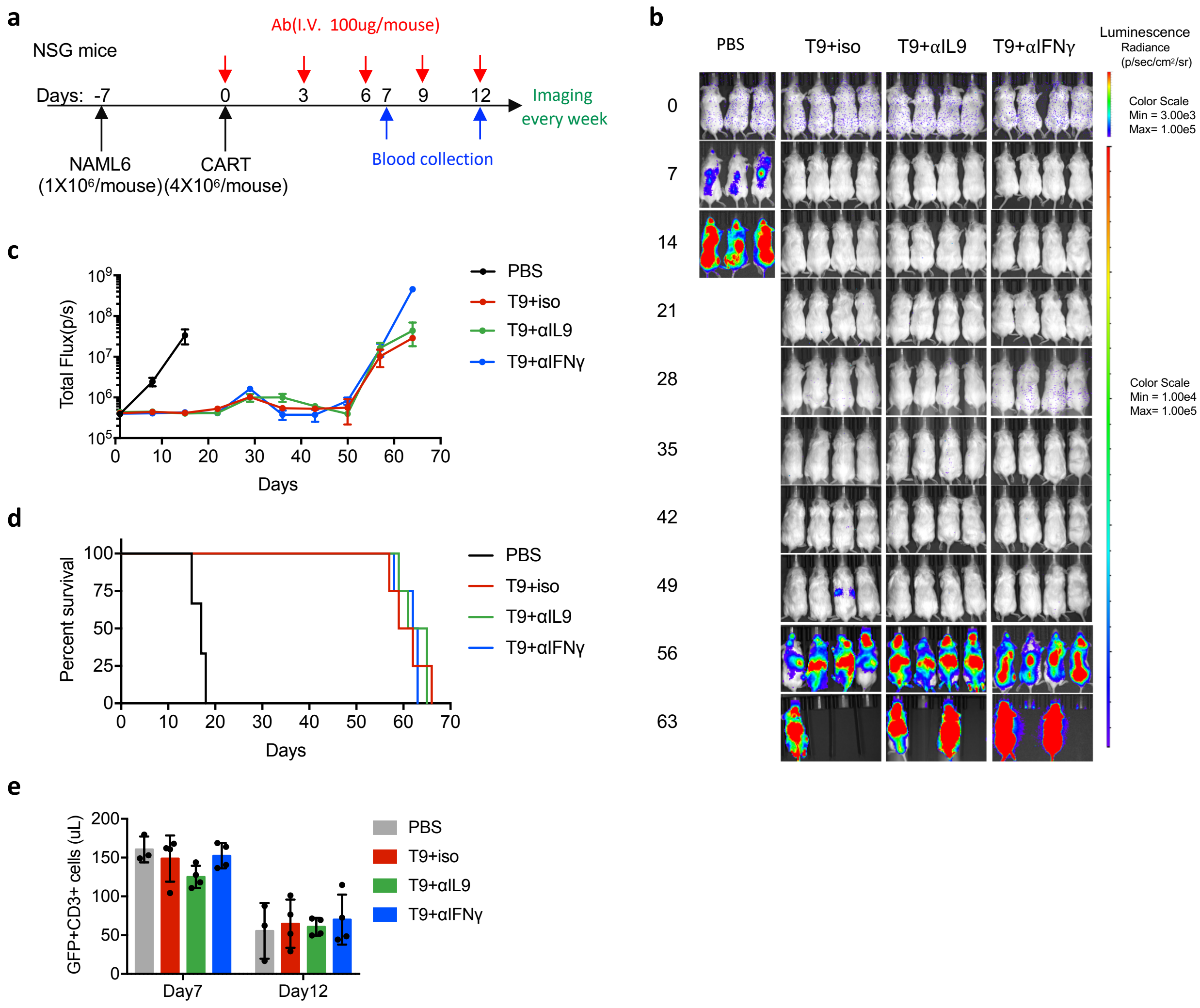

Supplementary Figure 9. IL9 and IFNγ do not play a major role in T9 CAR-T cell-mediated antitumor effects in vivo. (a) NSG mice were intravenously injected with 1×10^6 NALM6 cells. Seven days later, mice were randomly assigned to 4 groups and were infused intravenously with 4×10^6 $CD3^+$ CAR-T cells. Cytokine-neutralizing antibodies were infused introvenously every 3 days from day 0 to day 12 at a dose of 100 µg/mouse/time. **(b)** Tumor burden measured by bioluminescence at indicated days since CAR-T cell infusion. **(c)** Tumor burden (total flux) quantified by photons/s in mice treated with PBS or CAR-T cells at indicated days since CAR-T cell infusion. **(d)** Kaplan-Meyer plot showing mouse survival. (**e)** Total number of $CD3^+$ CAR-T cells in blood of treated mice at days 7 and 12 after CAR-T infusion.

n = 3 mice in PBS group, n = 4 in other groups. Data are presented as mean ± SD. Source data are provided as a Source Data file.

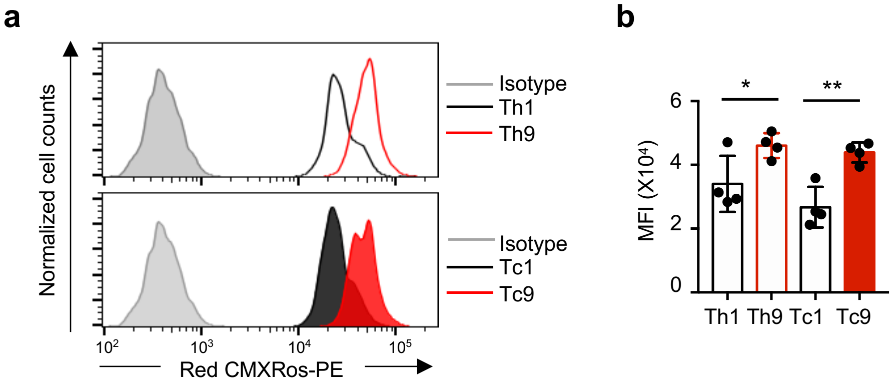

Supplementary Figure 10. **(a)** Representative flow plots showing frequency of Red CMXRos^{+} cells 8 days after CAR-T transfer. Cells were pre-gated for GFP^{+}CD3^{+}CD8^{+} or GFP^{+}CD3^{+}CD4^{+} T cells. (**b)** Summarized data of mean fluorescent intensity (MFI) for Red CMXRos staining (n = 4 mice). Data are presented as mean ± SD. *P < 0.05 and **P < 0.01, two-sided Student's *t*-test. Source data are provided as a Source Data file.

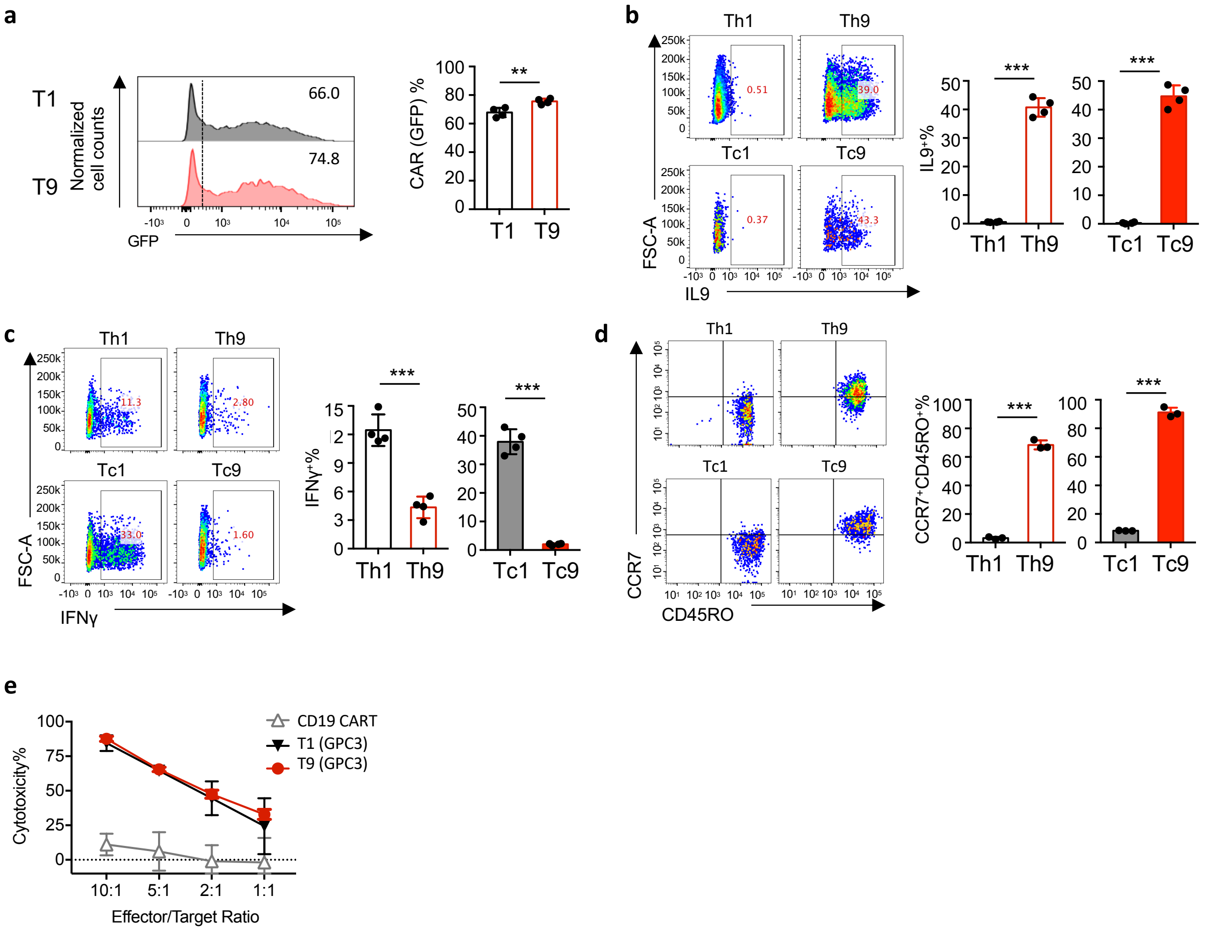

Supplementary Figure 11. Antitumor effects of GPC3 CAR-T cells. GPC3 CAR-T cells were generated and expanded for 14 days under Th1- or Th9-polarization condition. (**a**) CAR (GFP) expression (in percentage) was calculated in $CD3^+$ cell population (n = 4 donors). (**b**) Representative flow plots (left panels) and summarized data (right panels; n = 4 donors) showing IL9 expression in GPC3 CAR-T cells. Cells were pre-gated for $GFP^+CD3^+CD8^+$ or $GFP^+CD3^+CD4^+$ T cells. (**c**) Representative flow plots (left panels) and summarized data (right panels; n = 4 donors) showing IFNγ expression in CAR-T cells. Cells were pre-gated for $GFP^+CD3^+CD8^+$ or $GFP^+CD3^+CD4^+$ T cells. (**d**) Representative flow plots and pie charts (n = 3 donors) showing CCR7 or CD45RO expression on CAR-T cells. (**e**) Percentage of specific lysis of CAR-T cells against target tumor cells (HepG2) at indicated E:T ratios determined by flow

cytometry in 24 hour culture. Experiments were performed with three biological replicates and data (n = 3) shown are representative of two independent experiments. Data are presented as mean ± SD. *P < 0.05, **P < 0.01, and ***P < 0.001, two-sided Student's *t*-test. Source data are provided as a Source Data file.
