# Supplementary material for: Enhanced CAR-T activity against established tumors by polarizing human T cells to secrete interleukin-9
Source: Nat Commun. 2020 Nov 19;11:5902. doi: 10.1038/s41467-020-19672-2 (PMC7677397; doi:10.1038/s41467-020-19672-2)
Supplement: Supplementary file 3 — Reporting Summary [file 41467_2020_19672_MOESM3_ESM.pdf]

nature research

Corresponding author(s): Qing Yi

Last updated by author(s): Sep 25, 2020

Reporting Summary

Nature Research wishes to improve the reproducibility of the work that we publish. This form provides structure for consistency and transparency in reporting. For further information on Nature Research policies, see Authors & Referees and the Editorial Policy Checklist .

Statistics

For all statistical analyses, confirm that the following items are present in the figure legend, table legend, main text, or Methods section.

n/a	Confirmed	
☐	☒	The exact sample size (*n*) for each experimental group/condition, given as a discrete number and unit of measurement
☐	☒	A statement on whether measurements were taken from distinct samples or whether the same sample was measured repeatedly
☐	☒	The statistical test(s) used AND whether they are one- or two-sided<br>*Only common tests should be described solely by name; describe more complex techniques in the Methods section.*
☒	☐	A description of all covariates tested
☒	☐	A description of any assumptions or corrections, such as tests of normality and adjustment for multiple comparisons
☐	☒	A full description of the statistical parameters including central tendency (e.g. means) or other basic estimates (e.g. regression coefficient) AND variation (e.g. standard deviation) or associated estimates of uncertainty (e.g. confidence intervals)
☐	☒	For null hypothesis testing, the test statistic (e.g. *F*, *t*, *r*) with confidence intervals, effect sizes, degrees of freedom and *P* value noted<br>*Give P values as exact values whenever suitable.*
☒	☐	For Bayesian analysis, information on the choice of priors and Markov chain Monte Carlo settings
☒	☐	For hierarchical and complex designs, identification of the appropriate level for tests and full reporting of outcomes
☒	☐	Estimates of effect sizes (e.g. Cohen's *d*, Pearson's *r*), indicating how they were calculated

Our web collection on statistics for biologists contains articles on many of the points above.

Software and code

Policy information about availability of computer code

Data collection	BD FACSDiva software 8.1
Data analysis	Graphpad Prism v7.04, FlowJo 10.4, SOAPnuke v1.5.2, Bowtie2 v2.2.5, RSEM v1.2.12, gplots 4.0.2, DESeq2, GSEA v4.0.3

For manuscripts utilizing custom algorithms or software that are central to the research but not yet described in published literature, software must be made available to editors/reviewers. We strongly encourage code deposition in a community repository (e.g. GitHub). See the Nature Research guidelines for submitting code & software for further information.

Data

Policy information about availability of data

All manuscripts must include a data availability statement. This statement should provide the following information, where applicable:

- Accession codes, unique identifiers, or web links for publicly available datasets
- A list of figures that have associated raw data
- A description of any restrictions on data availability

The source data underlying Figs. 1a-e, g, 2a c i, 3d f h, 4e d f-h, 5b d f, 6b-c e-f, 7b-c e g j l n and Supplementary Figs. 1, 3a, 4b, 5b-g, 6b-c, 7c-e, 8c-e, 9b, 10a-e are provided as Source Data file. RNA-Seq data are available at NCBI's functional genomic data repository Gene Expression Omnibus (GEO) under accession code GSE156075. Gene set for GSEA analysis including KEGG_APOPTOSIS, GO_CELL_CYCLE_CHECKPOINT, GO_CELL_CYCLE_DNA_REPLICATION, GO_REGULATION_OF_CELL_CYCLE_G2_M_PHASE_TRANSITION, GSE11057_EFF_MEM_VS_CENT_MEM_CD4_TCELL_DN, GSE26928_EFF_MEM_VS_CENTR_MEM_CD4_TCELL_DN, GSE11057_EFF_MEM_VS_CENT_MEM_CD4_TCELL_UP, GSE26928_EFF_MEM_VS_CENTR_MEM_CD4_TCELL_UP, are available from The Molecular Signature Database (MSiDB). All the other data supporting the findings of this study are available within the article, supplementary information, source files, and from the corresponding author upon reasonable request. Source data are provided with this paper.

Field-specific reporting

Please select the one below that is the best fit for your research. If you are not sure, read the appropriate sections before making your selection.

[x] Life sciences [ ] Behavioural & social sciences [ ] Ecological, evolutionary & environmental sciences

For a reference copy of the document with all sections, see nature.com/documents/nr-reporting-summary-flat.pdf

Life sciences study design

All studies must disclose on these points even when the disclosure is negative.

Sample size	Sample size was determined to be adequate based on the magnitude and consistency of measurable differences between groups.
Data exclusions	No data were excluded.
Replication	All attempts at data replication in which images were acquired were considered successfully.
Randomization	Data was collected randomly. For in vivo mouse xenograft experiments, the mice were randomly grouped prior to be treated.
Blinding	The investigators were blinded to allocation during experiments and outcome assessment.

Reporting for specific materials, systems and methods

We require information from authors about some types of materials, experimental systems and methods used in many studies. Here, indicate whether each material, system or method listed is relevant to your study. If you are not sure if a list item applies to your research, read the appropriate section before selecting a response.

Materials & experimental systems

n/a	Involved in the study	
[ ]	[x]	Antibodies
[ ]	[x]	Eukaryotic cell lines
[x]	[ ]	Palaeontology
[ ]	[x]	Animals and other organisms
[ ]	[x]	Human research participants
[x]	[ ]	Clinical data

Methods

n/a	Involved in the study	
[x]	[ ]	ChIP-seq
[ ]	[x]	Flow cytometry
[x]	[ ]	MRI-based neuroimaging

Antibodies

Antibodies used

Flow antibodies for CD3 (OKT3, Cat#317344, 1:500), CD4 (OKT4, Cat#317438, 1:500), CD8 (SK1, Cat#344724, 1:500), CCR7 (G043H7, Cat#353214, 1:500), CD45RO (UCHL1, Cat#304244, 1:500), TIM3 (F38-2E2, Cat#345006, 1:500), IL9 (MH9A4, Cat#507614, 1:500), IL2 (MQ1-17H12, Cat#500342, 1:500) were purchased from BioLegend. Antibodies for IFN-γ (B27, Cat#MHCIFG04, 1:500), PD1(EBIOJ105, Cat#12-2799-42, 1:500), and LAG3 (3DS223H, Cat#15-2239-42, 1:500) were purchased from eBioscience.

Granzyme B (GB11, Cat#561142, 1:500) were purchased from BD Biosciences.

IFN-γ-neutralizing antibody (clone B133.5, Cat#BE0235, 10mg/ml) was from BioXcell.

phospho-c-Jun(Ser73)(D47G9, Cat#3270, 1:1000), c-Jun((60A8, Cat#9165, 1:1000) and beta-Actin(13E5, Cat#4970, 1:2000) were from CellSignal.

Validation

CD3 Supplier webpage – proved to be working in flow cytometry analysis of Human peripheral lymphocytes. https://www.biolegend.com/en-us/products/brilliant-violet-421-anti-human-cd3-antibody-11976.

CD4 Supplier webpage – proved to be working in flow cytometry analysis of Human peripheral lymphocytes. https://www.biolegend.com/en-us/products/brilliant-violet-605-anti-human-cd4-antibody-7820.

CD8 Supplier webpage – proved to be working in flow cytometry analysis of Human peripheral lymphocytes. https://www.biolegend.com/en-us/products/alexa-fluor-700-anti-human-cd8-antibody-9062.

CCR7 Supplier webpage – proved to be working in flow cytometry analysis of Human peripheral lymphocytes. https://www.biolegend.com/en-us/products/apc-anti-human-cd197-ccr7-antibody-7536.

CD45RO Supplier webpage – proved to be working in flow cytometry analysis of Human peripheral lymphocytes. https://www.biolegend.com/en-us/products/pe-anti-human-cd45ro-antibody-858.

TIM3 Supplier webpage – proved to be working in flow cytometry analysis of Human peripheral lymphocytes. https://www.biolegend.com/en-us/products/pe-anti-human-cd366-tim-3-antibody-6121.

IL9 Supplier webpage – proved to be working in flow cytometry analysis of Human peripheral lymphocytes. https://

www.biolegend.com/en-us/products/apc-anti-human-il-9-antibody-18363
IL2 Supplier webpage – proved to be working in flow cytometry analysis of Human peripheral lymphocytes. https://www.biolegend.com/en-us/products/apc-cyanine7-anti-human-il-2-antibody-9088.
LAG3 Supplier webpage – proved to be working in flow cytometry analysis of Human peripheral lymphocytes. https://www.thermofisher.com/antibody/product/15-2239-42.html?CID=AFLLO-15-2239-42.
IFN-γ(B27) Proved to be working in flow cytometry analysis Bratke et al., 2005.
Granzyme B Supplier webpage – proved to be working in flow cytometry analysis of Human peripheral lymphocytes. https://www.bdbiosciences.com/us/applications/research/t-cell-immunology/regulatory-t-cells/intracellular-markers/cytokines-and-chemokines/human/pe-mouse-anti-human-granzyme-b-gb11/p/561142.
PD1 Supplier webpage – proved to be working in flow cytometry analysis of Human peripheral lymphocytes. https://www.thermofisher.com/antibody/product/CD279-PD-1-Antibody-clone-eBioJ105-J105-Monoclonal/12-2799-42.
IFN-γ-neutralizing antibody (clone B133.5) Supplier webpage – proved to be working in in-vitro IFNγ neutralization. https://bxcell.com/product/anti-h-ifn-gamma/.
phospho-c-Jun(Ser73) Proved to be working in western blotting analysis in Su et al., 2016.
c-Jun Supplier webpage – proved to be working in western blotting analysis. https://www.cellsignal.com/products/primary-antibodies/c-jun-60a8-rabbit-mab/9165?Ntk=Products&Ntt=9165.
beta-Actin Supplier webpage – proved to be working in western blotting analysis. https://www.cellsignal.com/products/primary-antibodies/b-actin-13e5-rabbit-mab/4970?Ntk=Products&site-search-type=Products&N=4294956287&Ntt=beta-actin+13e5&fromPage=plp.

Eukaryotic cell lines

Policy information about cell lines

Cell line source(s): 293T, HepG2, K562 and NALM6 cell lines were purchased from ATCC.

Authentication: None of the cell lines used were authenticated because they were derived from ATCC

Mycoplasma contamination: All cell lines were tested negative for mycoplasma contamination.

Commonly misidentified lines (See ICLAC register): No commonly misidentified cell lines were used.

Animals and other organisms

Policy information about studies involving animals; ARRIVE guidelines recommended for reporting animal research

Laboratory animals: 6-8 week male immunodeficient NSG (Stock No: 005557) mice were purchased from The Jackson Laboratory. All mice were kept under clean conditions in 12/12 light/dark cycle, 65-75°F and 40-60% humidity.

Wild animals: This study does not involve wild animals.

Field-collected samples: This study does not involve field-collected samples

Ethics oversight: All experiments complied with protocols were approved by the Institutional Animal Care and Use Committee of the Lerner Research Institute of Cleveland Clinic and the Houston Methodist Research Institute.

Note that full information on the approval of the study protocol must also be provided in the manuscript.

Human research participants

Policy information about studies involving human research participants

Population characteristics: N/A

Recruitment: N/A

Ethics oversight: Human T cells from healthy volunteer donors were purchased from Gulf Coast Regional Blood Center in Houston. All experiments complied with protocols were approved by Institutional Review Board at the Lerner Research Institute of Cleveland Clinic and the Houston Methodist Research Institute.

Note that full information on the approval of the study protocol must also be provided in the manuscript.

Flow Cytometry

Plots

Confirm that:

- [x] The axis labels state the marker and fluorochrome used (e.g. CD4-FITC).
- [x] The axis scales are clearly visible. Include numbers along axes only for bottom left plot of group (a 'group' is an analysis of identical markers).
- [x] All plots are contour plots with outliers or pseudocolor plots.
- [x] A numerical value for number of cells or percentage (with statistics) is provided.

Methodology

Sample preparation	Sample preparation listed in Methods.
Instrument	Samples were analyzed on LSRFortessa flow cytometer (BD Biosciences)
Software	Data was collected with the BD FACSDiva software and analyzed wit the BD FlowJo V10 software.
Cell population abundance	More than 80%.
Gating strategy	Debris was first excluded by a morphology gate based on FSC-A and SSC-A. Then, we gated on GFP+CD3+CD4+ and GFP+CD3 +CD8+ cells for further analysis.

- [x] Tick this box to confirm that a figure exemplifying the gating strategy is provided in the Supplementary Information.
